# Comparative Aerosol and Surface Stability of SARS-CoV-2 Variants of Concern

**DOI:** 10.3201/eid2905.221752

**Published:** 2023-05

**Authors:** Trenton Bushmaker, Claude Kwe Yinda, Dylan H. Morris, Myndi G. Holbrook, Amandine Gamble, Danielle Adney, Cara Bushmaker, Neeltje van Doremalen, Robert J. Fischer, Raina K. Plowright, James O. Lloyd-Smith, Vincent J. Munster

**Affiliations:** National Institute of Allergy and Infectious Diseases, National Institutes of Health, Hamilton, Montana, USA (T. Bushmaker, C.K. Yinda, M.G. Holbrook, D. Adney, N. van Doremalen, R.J. Fischer, V.J. Munster);; Montana State University, Bozeman, Montana, USA (T. Bushmaker, R.K. Plowright);; University of California, Los Angeles, California, USA (D.H. Morris, A. Gamble, J.O. Lloyd-Smith);; Bitterroot Health—Daly Hospital, Hamilton (C. Bushmaker)

**Keywords:** COVID-19, coronavirus disease, SARS-CoV-2, severe acute respiratory syndrome coronavirus 2, viruses, respiratory infections, zoonoses, aerosol, environmental stability, transmission, variants of concern, Omicron, United States

## Abstract

SARS-CoV-2 transmits principally by air; contact and fomite transmission may also occur. Variants of concern are more transmissible than ancestral SARS-CoV-2. We found indications of possible increased aerosol and surface stability for early variants of concern, but not for the Delta and Omicron variants. Stability changes are unlikely to explain increased transmissibility.

Since the initial emergence of SARS-CoV-2 (lineage A), new lineages and variants have emerged (*1*), typically replacing previously circulating lineages. The World Health Organization has designated 5 virus variants as variants of concern (VOCs) (*2*). To assess whether the transmission advantage of new VOCs might have arisen partly from changes in aerosol and surface stability, we compared them directly with a lineage A ancestral virus (WA1 isolate).

## The Study

We evaluated the stability of SARS-CoV-2 variants in aerosols and on high-density polyethylene (to represent a common surface) and estimated their decay rates by using a Bayesian regression model (Appendix). We generated aerosols (<5 μm) containing SARS-CoV-2 with a 3-jet Collison nebulizer and fed them into a Goldberg drum to create an aerosolized environment (Video), using an initial virus stock of 10^5.75^–10^6^ 50% tissue-culture infectious dose (TCID_50_) per mL. To measure surface stability, we deposited 50 μL containing 10^5^ TCID_50_ of virus onto polypropylene.

**Video:** Goldberg drum used with 3-jet Collison nebulizer to create an aerosolized environment comparing stability of SARS-CoV-2 variants of concern

For aerosol stability, we directly compared the exponential decay rate of different SARS-CoV-2 isolates (Table) by measuring virus titer at 0, 3, and 8 hours; the 8-hour time point was chosen through modeling to maximize information on decay rate given the observed 3-hour decay. We performed experiments as single runs (0-to-3 or 0-to-8 hours) and collected samples at start and finish to minimize virus loss and humidity changes from repeat sampling. We conducted all runs in triplicate. To estimate quantities of sampled virus, we analyzed air samples collected at 0, 3, or 8 hours postaerosolization by quantitative reverse transcription PCR for the SARS-CoV-2 envelope (E) gene to quantify the genome copies within the samples. To determine the remaining concentration of infectious SARS-CoV-2 virions, we titrated samples on standard Vero E6 cells. To check robustness, we also titrated the samples on 2 Vero E6 TMPRSS2-expressing lines, yielding similar results (Appendix). We estimated exponential decay of infectious virus relative to the amount of remaining genome copies to account for particle settling and other physical loss of viruses, although we also estimated decay rates from uncorrected titration data as a robustness check, which yielded similar results (Appendix).

**Table:** SARS-CoV-2 isolates used in study of comparative stability and their observed aerosol and surface half-lives*

SARS-CoV-2 isolate	WHO label	PANGO label	GISAID/GenBank accession no.	Aerosol half-life, h	Surface half-life, h
hu/USA/CA_CDC_5574/2020		A	MN985325.1	3.2 9 (2.33–4.98)	4.82 (4.23–5.49)
hCoV-19/USA/MT-RML-7/2020		B.1	MW127503.1	3.99 (2.73–7.2)	5.16 (4.48–5.96)
hCoV-19/England/204820464/2020	Alpha	B.1.1.7	EPI_ISL_683466	6.13 (3.14–27.5)	5.13 (4.59–5.74)
hCoV-19/USA/MD-HP01542/2021	Beta	B.1.351	EPI_ISL_890360	5.13 (3.16–12.3)	5.73 (5.01–6.72)
hCoV-19/USA/KY-CDC-2-4242084/2021	Delta	B.1.617.2	EPI_ISL_1823618	3.12 (2.29–4.73)	4.38 (3.48–5.65)
hCoV-19/USA/WI-WSLH-221686/2021	Omicron	B.1.1.529	EPI_ISL_7263803	2.15 (1.35–4.04)	3.58 (2.88–4.47)

*The half-life of the aerosols or on surface is presented as posterior median value with posterior quantiles. WHO, World Health Organization.

**Figure 1:**
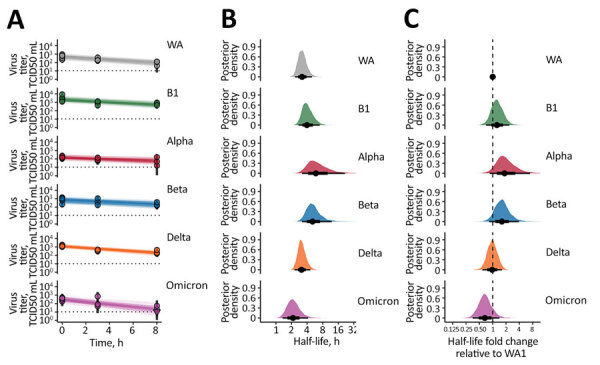
SARS-CoV-2 variant exponential decay in aerosolized form and corresponding half-lives. A) Regression lines representing predicted exponential decay of log10 virus titer over time compared with measured (directly inferred) virus titers. Points with black bars show individually estimated titer values (point: posterior median titer estimate; bar: 95% credible interval). Points at 3 hours and 8 hours are shifted up or down by the physical/noninactivation change in viral material estimated from quantitative reverse transcription PCR data (Appendix), to enable visual comparison with predicted decay (which reflects only inactivation effects). Semitransparent lines show random draws from the joint posterior distribution of the exponential decay rate and the drum run intercept (virus titer at t = 0); this visualizes the range of plausible decay patterns for each experimental condition. We performed 50 random draws and then plotted 1 line per draw for each drum run, yielding 300 plotted lines per variant. B) Inferred virus half-lives by variant, plotted on a logarithmic scale. Density plots show the shape of the posterior distribution. Dots show the posterior median half-life estimate and black lines show a 68% (thick) and 95% (thin) credible interval. C) Inferred ratio of variant virus half-lives to that of WA1 (fold-change), plotted on a logarithmic scale and centered on 1 (no change, dashed line). Dot shows the posterior median estimate and black lines show a 68% (thick) and 95% (thin) credible interval. TCID50, 50% tissue culture infectious dose.

We recovered viable SARS-CoV-2 virus from the drum for all VOCs (Figure 1, panel A). The quantity of viable virus decayed exponentially over time (Figure 1, panel B). The half-life of the ancestral lineage WA1 in aerosols (posterior median value [2.5%–97.5% posterior quantiles]) was 3.20 (2.33–4.98) hours. The B.1, Alpha, and Beta viruses appeared to have longer half-lives than WA1: 3.99 (2.73–7.20) hours for B.1, 6.13 (3.14–27.5) hours for Alpha, and 5.13 (3.16–12.3) hours for Beta. The half-life of Delta was similar to that of WA1: 3.12 (2.29–4.73) hours. The Omicron (BA.1) variant displayed a similar or decreased half-life compared with WA1: 2.15 (1.35–4.04) hours (Figure 1, panel B). To better quantify the magnitude and certainty of the change, we computed the posterior of the ratio for variant half-life to WA1 half-life for each variant (Figure 1, panel C). Estimated ratios were 1.25 (0.701–2.48) for B.1, 1.88 (0.859–8.75) for Alpha, 1.6 (0.838–4.01) for Beta, 0.978 (0.571–1.63) for Delta, and 0.659 (0.35–1.37) for Omicron. That is, initial spike protein divergence from WA1 (heuristically quantified by the number of amino acid substitutions) appeared to produce increased relative stability, but further evolutionary divergence reverted stability back to that of WA1, or even below it (Figure 1, panel C; Appendix Figures 1, 2).

**Figure 2:**
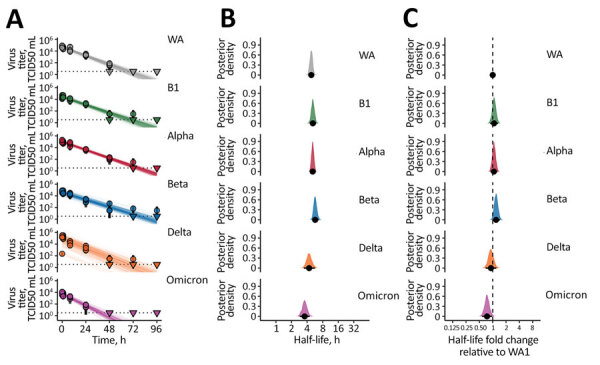
SARS-CoV-2 variant exponential decay on an inert surface and corresponding half-lives. A) Regression lines representing predicted exponential decay of log10 virus titer over time compared with measured (directly inferred) virus titers. Semitransparent lines show random draws from the joint posterior distribution of the virus exponential decay rate and the sample intercepts (virus titers at t = 0). We performed 50 random draws and then plotted 6 random initial titers per draw for each variant, yielding 300 plotted lines per variant. We chose a new group of 6 random initial titers for each new draw-variant pair. Points with black bars show individually estimated titer values (point: posterior median titer estimate; bar: 95% credible interval). Samples with no positive titration wells are plotted as triangles at the approximate LOD (dotted horizontal line). B) Inferred virus half-lives by variant, plotted on a logarithmic scale. Density plots show the shape of the posterior distribution. Dots show the posterior median half-life estimate and black lines show a 68% (thick) and 95% (thin) credible interval. C) Inferred ratio of variant virus half-lives to that of WA1 (fold-change), plotted on a logarithmic scale and centered on 1 (no change, dashed line). Dot shows the posterior median estimate and black lines show a 68% (thick) and 95% (thin) credible interval. TCID50, 50% tissue culture infectious dose.

Next, we investigated surface stability of VOCs compared with the ancestral variant on polyethylene. Again, all variants exhibited exponential decay (Figure 2, panel A). We found a half-life of 4.82 (4.23–5.49) hours for WA1, similar to our previous estimates (Figure 2, panel B) (*3*). Early VOCs had slightly longer half-lives: 5.16 (4.48–5.96) hours for B.1, 5.13 (4.59–5.74) hours for Alpha, and 5.73 (5.01–6.72) hours for Beta (Figure 2, panel B). As with aerosols, Delta had a half-life similar to WA1 of 4.38 (3.48–5.65) hours and Omicron had a somewhat shorter half-life of 3.58 (2.88–4.47) hours (Figure 2, panel B). We again calculated posterior probabilities for the half-life ratios relative to WA1 (Figure 2, panel C). B.1 had a half-life ratio to WA1 of 1.07 (0.876–1.32), Alpha a half-life ratio of 1.07 (0.896–1.27), and Beta a half-life ratio of 1.19 (0.988–1.46). The ratios for Delta and Omicron were 0.912 (0.694–1.21) and 0.744 (0.578–0.965).

In both aerosol and surface results, the posterior 95% credible intervals for most ratios overlap 1. Experimental noise could possibly explain the apparent trend toward increased stability for B.1, Alpha, and Beta, although the clear bulk of posterior probability mass indicates greater half-lives. Conversely, the posterior ratios indicate clearly that Delta and Omicron are not markedly more stable than WA1 and might be less stable (particularly Omicron and particularly on surfaces).

## Conclusions

Several studies have analyzed the stability of SARS-CoV-2 on surfaces or in aerosols in a Goldberg rotating drum (*3*–*7*). Most have focused on the duration over which infectious virus could be detected. In this study, we paired a model-optimized experimental design with Bayesian hierarchical analysis to systematically measure virus half-life across 6 SARS-CoV-2 variants and directly estimate relative half-lives with full error propagation. We found a small initial increase in aerosol stability from ancestral WA1 to the B.1, Alpha, and Beta variants, with some statistical uncertainty. However, we found that Delta has a half-life similar to that of WA1 and that Omicron likely has a shorter one. In surface measurements, the VOCs followed the same pattern of relative stability, confirming that the overall stability of SARS-CoV-2 variants is determined by similar factors in aerosols and on surfaces (*8*). Divergent results on the aerosol and surface stability of VOCs have been reported (*7*,*8*).

Our study suggests that aerosol stability is likely not a major factor driving the increase in transmissibility observed with several VOCs (*9*,*10*). The early rise in stability for B.1 and its descendants Alpha and Beta might have arisen incidentally from selection for other viral traits that favored higher transmission. Epidemiologic and experimental studies suggest that the window for transmission is typically relatively short (<1 hour), and thus a modest change in aerosol half-life would not have discernible epidemiologic effects (*11*). However, in specific contexts of enclosed spaces, it will remain vital to understand the temporal profile of transmission risks after the release of aerosols containing SARS-CoV-2 from an infected person. We conducted our experiments under laboratory conditions using tissue culture media, so biological factors potentially affecting decay (e.g., airway mucins and other components of airway-lining fluids) were not considered. Novel approaches studying aerosol microenvironments have reported initial rapid loss of SARS-CoV-2 infectiousness in the seconds after aerosolization (*12*); our work only addresses SARS-CoV-2 decay and stability over longer timescales, after the initial deposition loss has occurred.

Whereas evolutionary selection for previous variants favored high transmission among immunologically naive humans (*13*), since late 2021, global population-level selection has favored antigenic change (*14*) and the consequent ability to transmit among nonnaive persons. Our findings suggest that increased transmissibility through antigenic evolution might come at a tolerable cost to the virus in environmental stability. Overall, the differences in environmental stability among different VOCs, in aerosols or on surfaces, are unlikely to be driving variant population-level epidemiology.

Appendix Additional information about comparative aerosol and surface stability of SARS-CoV-2 variants of concern
