# Supplementary figures and images for: Comparative Aerosol and Surface Stability of SARS-CoV-2 Variants of Concern
Source: Emerg Infect Dis. 2023 May;29(5):1033–7. doi: 10.3201/eid2905.221752 (PMC10124653; doi:10.3201/eid2905.221752)

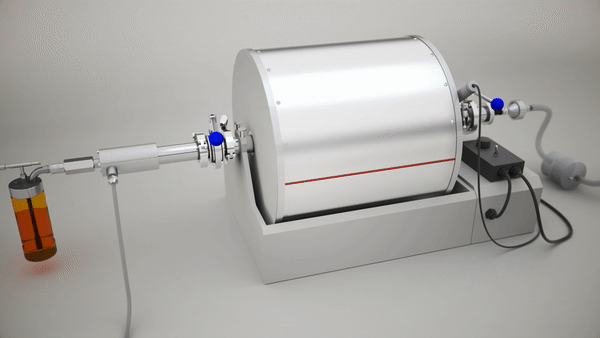

Supplement: Supplementary file 1 [file 22-1752-V.gif]
