# Supplementary material for: Comparative Aerosol and Surface Stability of SARS-CoV-2 Variants of Concern
Source: Emerg Infect Dis. 2023 May;29(5):1033–7. doi: 10.3201/eid2905.221752 (PMC10124653; doi:10.3201/eid2905.221752)
Supplement: Appendix — Additional information about comparative aerosol and surface stability of SARS-CoV-2 variants of concern [file 22-1752-Techapp-s1.pdf]

Article DOI: https://doi.org/10.3201/eid2905.221752

EID cannot ensure accessibility for Supplemental Materials supplied by authors. Readers who have difficulty accessing supplementary content should contact the authors for assistance.

Comparative Aerosol and Surface Stability of SARS-CoV-2 Variants of Concern

Appendix

Experimental methods

Cells and viruses

We passaged SARS-CoV-2 strains once on Vero E6 cells maintained in DMEM supplemented with 10% FBS, 2 mM L-glutamine, 100 U/ml penicillin and 100 μg/ml streptomycin.

The CDC (Atlanta, USA) provided an isolate of the ancestral WA1 (PANGO lineage A) strain (hu/USA/CA_CDC_5574/2020, GenBank MN985325.1). We used a B.1 lineage virus (hCoV-19/USA/MT-RML-7/2020, GenBank MW127503.1, GISAID# EPI_ISL_591054) derived from a clinical specimen obtained from Bitterroot Health-Daly Hospital Hamilton, USA. We obtained the Alpha variant (PANGO B.1.1.7, hCoV-19/England/204820464/2020, BEI catalog # NR-54000, GISAID# EPI_ISL_683466) from BEI Resources, NIAID, NIH; virus contributed by Dr. Bassam Hallis. We obtained the Beta variant (PANGO B.1.351, hCoV-19/USA/MD-HP01542/2021, GISAID# EPI_ISL_890360) from Dr. Tulio de Oliveira and Dr. Alex Sigal at the Nelson R. Mandela School of Medicine, UKZN. We obtained the Delta variant (PANGO B.1.617.2, hCoV-19/USA/KY-CDC-2–4242084/2021, GISAID# EPI_ISL_1823618) from BEI resources. We obtained the Omicron variant (PANGO B.1.1.529 or BA.1, hCoV-

19/USA/WI-WSLH-221686/2021, GISAID# EPI_ISL_7263803) from Drs. Peter Halfmann and Yoshihiro Kawaoka at the University of Wisconsin, Madison, USA (see also Main Text Table).

We propagated virus stocks in Vero E6 cells in DMEM supplemented with 2% fetal bovine serum (FBS), 2 mM L-glutamine, 100 U/ml penicillin and 100 µg/mL streptomycin. We harvested stocks between Day 4 and day 6, depending on the cytopathic effect. We collected supernatant was collected, centrifuged it at 1200 rpm for 8 minutes at room temperature, and froze it at −80°C. To confirm that virus isolate genomes were identical to those deposited in GenBank and/or GISAID, we performed deep sequencing with the Illumina MiSeq system (300-cycle Nano kit, Illumina); no SNPs relative to the original patient sample sequence were detected. The data are presented in Appendix Figure 2.

Aerosol stability experiment

We generated small aerosols (<5 µm in diameter) using a 3-jet Collison nebulizer (CH Technologies) containing $10^{5.75}$–10^{6} $TCID_{50}$/mL in 10 mL of DMEM supplemented with 2% FBS. We fed the nebulized inoculum into a rotating Goldberg drum (Biaera Technologies) to create the aerosol environment. For all runs, the drum system was prepared by a 7 minutes loading of the drum and an additional 5 minutes of stabilization until a starting environment of 65% relative humidity (RH) and a temperature of 21–23°C was reached. We used a drum rotational velocity of 3 miles h^{-1} to overcome aerosol settling velocity and thus maintain particle suspension.

We performed three independent replicate drum runs for each target timepoint (3 h or 8 h) for each variant. For each run, we collected a $t = 0$ sample after equilibration and then a final (t = 3 h or 8 h) sample. We collected samples by drawing air at 6 L min^{-1} for 30 s onto a 47 mm gelatin filter (Sartorius). We dissolved filters in 10 mL of DMEM containing 10% FBS at 37°C, and froze the resultant samples at −80°C until assessment.

Virus quantification

qPCR

We measured virus RNA in aerosol samples using qRT-PCR as previously described (*1*). In short, we used 140 µL of sample for RNA extraction with the QIAamp Viral RNA Kit (Qiagen) using the QIAcube HT automated system (Qiagen) with an elution volume of 150 µL. We used the E gene assay for SARS-CoV-2 RNA (*1*) with 5 µL of input RNA and the TaqMan Fast Virus One-Step Master Mix (Applied Biosystems), run on a QuantStudio 6 Flex Real-Time PCR System (Applied Biosystems). We ran 10-fold dilutions of SARS-CoV-2 E gene run-off transcripts and 10-fold dilutions with known genome copies in parallel to estimate sample copy numbers.

Titration

We determined infectious virus titers by endpoint titration. Results in the Main Text show titration on standard Vero E6 cells. For sensitivity analysis, we also titrated samples on Vero E6-TMPRSS2-T2A-ACE2 cells (BEI catalog # NR-54970), and on Vero-TMPRSSII-RML cells. We inferred $TCID_{50}$/mL values from titration data in a Bayesian framework as described below.

Surface stability experiment

We measured surface stability 15 mm polypropylene at 21–23°C and 40% RH. We deposited 50 µL of virus stock containing 10^5 $TCID_{50}$/mL (7–10 drops) on the surface of a disc. At predefined time-points, we sampled viable virus rinsing discs with 1 mL of Dulbecco's modified Eagle's medium (Sigma-Aldrich, St, Louis, MO) supplemented with 2% fetal bovine serum, 1 mM L-glutamine, 50 U/ml penicillin and 50 µg/mL streptomycin (2% DMEM). We froze samples at −80°C until titration. We performed three replicate experiments for each variant and determined infectious virus titers by endpoint titration as described above.

Bayesian inference methods

Conceptual overview

Building on our prior work (*2–4*), we inferred individual titers and virus half-lives in a Bayesian framework, modeling the positive or negative status of individual observed titration wells according to a Poisson single-hit process (*5*). This can then be used either to infer individual titers or to fit an exponential decay rate (equivalent, a half-life) to a set of samples taken at different timepoints. In the latter case, we jointly infer the decay rate and the individual titers, for maximally principled error propagation. The reason we also estimate individual titer values (without any assumptions about their relationship or the decay process) is that this allows us to check goodness-of-fit of the exponential decay model.

Notation

In the text that follows, we use the following mathematical notation.

Logarithms and exponentials

$log(x)$ denotes the logarithm base e of x (sometimes called $ln(x)$). We explicitly refer to the logarithm base 10 of x as $log_{10}(x)$. $exp(x)$ denotes e^x.

Probability distributions

The symbol $\sim$ denotes that a random variable is distributed according to a given probability distribution. So for example

$$X \sim Normal(0,1)$$

indicates that the random variable X is normally distributed with mean 0 and standard deviation 1.

We parameterize normal distributions as:

$$Normal(mean, standarddeviation)$$

We parameterize positive-constrained normal distributions (i.e., with lower limit 0) as:

$$PosNormal(mode, standarddeviation)$$

We parameterize Poisson distributions as:

$$Poisson(mean)$$

Titer inference

For both surface and aerosol samples, we estimated individual sample infectious virus titers directly from titration well data as previously described (*3*), using a weakly informative Normal prior on the true virus concentration v_i in units of $TCID_{50}$/0.1 mL (since well inocula were 0.1 mL):

$$v_i \sim Normal(3,3)$$

Surface half-life inference

Similarly, we inferred half-lives of infectious virus on surfaces using the method previously described in (*3*), which allows us to account for variation in initial virus deposition on individual coupons, among other sources of experimental error. We used the following priors.

Log half-lives $log(h_i)$ for each experimental condition i:

$$log(h_i) \sim Normal\big(log(5), log(4)\big)$$

Mean initial $log_{10}/0.1mL$ virus titers $\bar{v}_{0i}$ for each experimental condition i:

$$\bar{v}_{0i} \sim Normal(3,2)$$

Experiment-specific standard deviations σ_i of initial initial $\log_{10}$ titers v_{0ij} about the mean $\bar{v}_{0i}$ for each experimental condition i:

$$\sigma_i \sim PosNormal(0.4,0.3)$$

Aerosol half-life inference

To conduct aerosol half-life inference, we had to account for settling and re-suspension, drum sampling noise, other loss/gain of viral material unrelated to virus inactivation. We did this by incorporating qPCR measurements of the virus genome quantity in our samples.

qPCR adjustment for changes in sampled viral material

Let $f(t) = \frac{V(t)}{N(t)}$ denote the ratio of infectious virus (in units of $TCID_{50}$ per unit volume) to virus RNA (in units of genome copies or qPCR copying events (i.e., 2^{CT}) for the aerosols in the drum. If infectious virus decays exponentially, then:

$$log_{10}[f(t)] = log_{10}[f(0)] - \lambda t$$

where λ is the exponential decay rate in $\log_{10}$ infectious virus per unit time.

So in a sample with $N(t)$ genome copies taken at time t, we expect to find a titer of:

$$log_{10}[V(t)] = log_{10}[f(t)N(t)] = log_{10}[f(0)] - \lambda t + log_{10}[N(t)]$$

But since $f(0) = \frac{V(0)}{N(0)}$, $log_{10}[(f(0)] = log_{10}[V(0)] - log_{10}[N(0)]$, so this is equivalent to:

$$log_{10}[V(t)] = log_{10}[V(0)] - \lambda t + log_{10}[N(t)] - log_{10}[N(0)]$$

In other words, it is equal to the naive prediction (without qPCR data on non-inactivation loss/gain of virus) minus a correction term reflecting the measured log_{10} fold change in virus RNA (e.g., non-inactivation loss of infectious virus), which we call L:

$$L = log_{10}[N(t)] - log_{10}[N(0)]$$

Notice that if L is negative—$N(t) < N(0)$—we expect to see an additional decrease in sample titer beyond that predicted by actual virus inactivation, and so we will measure a longer

half-life than we would have had we not corrected for changing quantities of viral material (infectious or not) in the sample.

For each drum run j of experimental condition i, we estimated L_{ij} for sample $1ij$ (taken at $t = 3h$ or $t = 8h$) by the change in sample CT values C_{ij} relative to the initial $t = 0h$ sample $0ij$:

$$L_{ij} = -log_{10}(2)\left[C_{1ij} - C_{0ij}\right]$$

We also considered defining L in terms of the change in estimated genome copy numbers n, i.e.:

$$L_{ij} = log_{10}\left[n_{1ij}\right] - log_{10}\left[n_{0ij}\right]$$

but these L values were sufficiently similar to those obtained from CT values that we did not rerun the analysis.

Titer prediction

For each drum run j, we then predicted the measured final infectious virus titer v_{1ij} given the $t = 0h$ measurement v_{0ij} as:

$$v_{1ij} = v_{0ij} + L_{ij} - \lambda_i t_{ij}$$

where t_{ij} is the timepoint for the second sample (3 h or 8 h) and λ_i is the exponential decay rate in $\log_{10}$ infectious virus per hour, calculated from the half-life as:

$$\lambda_i = \frac{log_{10}(2)}{h_i}$$

We modeled initial sampled titers v_{0ij} for each individual drum run j of experiment i as distributed about an inferred experiment-specific mean $\bar{v}_{0i}$, with an inferred experiment-specific standard deviation σ_i:

$$v_{0ij} \sim Normal(\bar{v}_{0i}, \sigma_i)$$

We modeled observed titration wells for both v_{0ij} and v_{1ij} according to the same Poisson single-hit process previously described and used to estimate individual titers and surface half-lives.

Prior distributions

We used the following prior distributions.

Log half-lives $log(h_i)$ for each experimental condition i:

$$log(h_i) \sim Normal\big(log(5), log(4)\big)$$

Mean initial virus titers $\bar{v}_{0i}$ (in units of $log_{10}TCID_{50}/0.1mL$ titrated sample):

$$\bar{v}_{0i} \sim Normal(2,2)$$

Standard deviations σ_i of individual initial titers v_{0ij} about the experiment mean $\bar{v}_{0i}$:

$$\sigma_i \sim PosNormal(0.4,0.2)$$

Prior predictive checks

We assessed appropriateness of prior distributions with prior predictive checks. These confirmed that the priors allowed for a wide range of plausible inactivation kinetics both for aerosol experiments (Figure 3) and for surface experiments (Figure 4).

We visualized prior predictive draws as possible decay lines, determined by a predicted $t = 0$ value and a predicted decay rate. For the aerosol experiments, each plotted line can be thought of as a prior prediction for a single drum run, since the estimated titers there represent a single timeseries with a shared $t = 0$ value (intercept). For the surface experiments, each line can be thought of as a prior prediction for a single measured titer, since each sample deposited on a surface has its own $t = 0$ value from which it decays until the time it is sampled.

To show the joint prior, we plotted multiple lines for each of the random posterior draws, one for each of several intercepts / timeseries. For the aerosol experiments, we plotted one line for each drum run. In the surface experiments, there is one intercept per titer; to keep the number of lines manageable, we plotted a random set of 6 intercept parameters from each experiment for each posterior draw. We resampled which intercepts to plot every draw, so many different sets of 6 are plotted for each experiment (one set for every plotted draw).

Computational methods

As previously described, we fit the models described above our data using Stan (*6*), which implements a No-U-Turn Sampler (*7*). We inferred all parameters jointly for all models. We ran 4 parallel Markov chains with 1000 iterations of warmup followed by 1000 sampling iterations, resulting in a total of 4000 posterior samples for each inference model. We assessed chain mixing and convergence by inspecting trace plots and confirming sufficient effective sample size and lack of divergent transitions.

We created visualizations and tables in R using ggplot2 (*8*), ggdist (*9*), and tidybayes (*10*).

Sensitivity analysis and robustness checks

Posterior predictive checks

To assess model goodness of fit, we performed posterior predictive checks. These are visualized identically to the prior predictive checks shown above. These predictive checks differ from the regression fits shown in the Main Text in that the intercepts of the lines plotted are random draws from the posterior predictive distribution (given by the inferred experiment-specific means and standard deviations) rather than the specific inferred values corresponding to individual actually-observed timeseries / titers. So the close match of the lines to the data here is a stronger test of model appropriateness. In particular, it shows that the hierarchical modeling of intercepts is capturing the degree of experimental variation well. Note that exponential decay

rates shown in the posterior checks *are* the posterior inferred values, as we estimated decay rates without hierarchy.

Alternative cell lines

Different VOC show different cell tropism and interactions with human proteases (*11*,*12*; T.P. Peacock et al., unpub. data, https://www.biorxiv.org/content/10.1101/2021.12.31.474653v2). For example, Delta appears to use TMPRSS2-mediated cell entry routes more than Omicron BA.1, and TMPRSS2-deletion affects in vitro Delta cell entry more than Omicron cell entry (*12*). Similarly, spike evolution may involve tradeoffs between protein stability and virion fusogenicity, and different VOC may have distinct stability / fusogenicity properties (*13*).

To ensure that our environmental stability results were not an artifact of an interaction between our choice of titration cell line and the cell entry properties of the VOC, we titrated aerosol samples on two additional cell lines, both Vero E6-derived lines modified to express TMPRSS2: Vero E6-TMPRSS2-T2A-ACE2 and Vero-TMPRSSII-RML. Figure 7 shows estimated half-lives by variant and cell line. Figure 8 shows estimated fold-changes in half-life relative to WA1 by variant and cell line.

To allow more detailed comparison with our main results, including assessment of model fit, we show versions of Main Text Figure 1 for the alternative cell lines below (Figures 9, 10), as well as corresponding posterior predictive checks (Figures 11, 12).

Estimates without qPCR adjustment

We also wished to ensure that our aerosol results were not an artifact of the qPCR adjustment used, so we estimated raw titration half-lives (i.e., half-life including non-inactivation loss of infectious material).

As above, we visualize estimated half-lives (Figure 13), estimated fold-changes in half-life relative to WA1 (Figure 14), and then plot versions of Main Text Figure 1 for each cell line: Vero E6, as in Main Text, Figure 15; Vero E6-TMPRSS2-T2A-ACE2, Figure 16, Vero-TMPRSSII-RML, Figure 17. The only difference is that here all plotted estimates are without qPCR adjustment for non-inactivation changes in viral material.

The same qualitative patterns persists in all cases, and in fact the statistical signal of increased half-life of B.1, Alpha, and Beta relative to WA1 is stronger for non-qPCR-adjusted half-lives than for qPCR-adjusted ones. As expected, estimated half-lives tend to be shorter, likely due to loss of viral material by processes other than inactivation (e.g., settling).

Further discussion

The principal difference between the drum and the surface experiments is that in the drum experiments we directly sample v_{0ij}, as this can be done non-destructively (where it cannot be done with an individual surface sample).

Note that the $t = 0h$ sample in the aerosol experiments occurs after a drum equilibriation period, and thus after any physical loss from that occurs during the aerosolization process and any rapid initial loss of infectious virus, as has been reported in other studies of aerosolized virus (*14*).

Except for very near-field airborne exposure (e.g., a person shouting in another's face), the transmission-relevant half-life of infectious virus in aerosols is the quasi-equilibrium half-life after any rapid initial loss has occurred. This later half-life is the one our experiment is designed to measure (note that our $t = 0h$ titers are much lower than our stock solutions, see Experimental methods: Aerosol stability experiment).

Similarly, it is important to note that real-world depositions in aerosols or onto surfaces may differ markedly in absolute quantity of infectious virus deposited. Here and in other studies, we use large initial quantities not because these are necessarily a realistic stand in for any or all

depositions (*15*), but rather because this enables maximally informative estimates of decay rates and half-lives. Since the decay process is approximately exponential, these rate estimates can be used for risk assessment for a wide range of deposition sizes.

Code and data

All code and data needed to reproduce our analyses is archived on Github (https://github.com/dylanhmorris/aerosol-stability-voc) and Zenodo (https://doi.org/10.5281/zenodo.7675574), and licensed for reuse, with appropriate attribution and citation.

References

1. Corman VM, Landt O, Kaiser M, Molenkamp R, Meijer A, Chu DK, et al. Detection of 2019 novel coronavirus (2019-nCoV) by real-time RT-PCR. Euro Surveill. 2020;25:2000045. PubMed https://doi.org/10.2807/1560-7917.ES.2020.25.3.2000045
2. Fischer RJ, Morris DH, van Doremalen N, Sarchette S, Matson MJ, Bushmaker T, et al. Effectiveness of N95 respirator decontamination and reuse against SARS-CoV-2 virus. Emerg Infect Dis. 2020;26:2253–5. PubMed https://doi.org/10.3201/eid2609.201524
3. Gamble A, Fischer RJ, Morris DH, Yinda CK, Munster VJ, Lloyd-Smith JO. Heat-treated virus inactivation rate depends strongly on treatment procedure: illustration with SARS-CoV-2. Appl Environ Microbiol. 2021;87:e0031421. PubMed https://doi.org/10.1128/AEM.00314-21
4. Morris DH, Yinda KC, Gamble A, Rossine FW, Huang Q, Bushmaker T, et al. Mechanistic theory predicts the effects of temperature and humidity on inactivation of SARS-CoV-2 and other enveloped viruses. eLife. 2021;10:e65902. PubMed https://doi.org/10.7554/eLife.65902
5. Brownie C, Statt J, Bauman P, Buczynski G, Skjolaas K, Lee D, et al. Estimating viral titres in solutions with low viral loads. Biologicals. 2011;39:224–30. PubMed https://doi.org/10.1016/j.biologicals.2011.06.007

6. Stan Development Team. The Stan core library. 2018 [cited 2023 Mar 4].

7. Hoffman MD, Gelman A, et al. The No-U-Turn sampler: adaptively setting path lengths in Hamiltonian Monte Carlo. J Mach Learn Res. 2014;15:1593–623.

8. Wickham H. ggplot2: Elegant graphics for data analysis. 2016 [cited 2023 Mar 4]. https://ggplot2.tidyverse.org

9. Kay M. ggdist: Visualizations of distributions and uncertainty. 2020 [cited 2023 Mar 4]. https://mjskay.github.io/ggdist/

10. Kay M. tidybayes: Tidy data and geoms for Bayesian models. 2020 [cited 2023 Mar 4]. https://mjskay.github.io/tidybayes/

11. McCallum M, Czudnochowski N, Rosen LE, Zepeda SK, Bowen JE, Walls AC, et al. Structural basis of SARS-CoV-2 Omicron immune evasion and receptor engagement. Science. 2022;375:864–8. PubMed https://doi.org/10.1126/science.abn8652

12. Meng B, Abdullahi A, Ferreira IATM, Goonawardane N, Saito A, Kimura I, et al.; CITIID-NIHR BioResource COVID-19 Collaboration; Genotype to Phenotype Japan (G2P-Japan) Consortium; Ecuador-COVID19 Consortium. Altered TMPRSS2 usage by SARS-CoV-2 Omicron impacts infectivity and fusogenicity. Nature. 2022;603:706–14. PubMed https://doi.org/10.1038/s41586-022-04474-x

13. Wolf KA, Kwan JC, Kamil JP. Structural dynamics and molecular evolution of the SARS-CoV-2 spike protein. MBio. 2022;13:e0203021. PubMed https://doi.org/10.1128/mbio.02030-21

14. Oswin HP, Haddrell AE, Otero-Fernandez M, Mann JFS, Cogan TA, Hilditch TG, et al. The dynamics of SARS-CoV-2 infectivity with changes in aerosol microenvironment. Proc Natl Acad Sci U S A. 2022;119:e2200109119. PubMed https://doi.org/10.1073/pnas.2200109119

15. Goldman E. Exaggerated risk of transmission of COVID-19 by fomites. Lancet Infect Dis. 2020;20:892–3. PubMed https://doi.org/10.1016/S1473-3099(20)30561-2

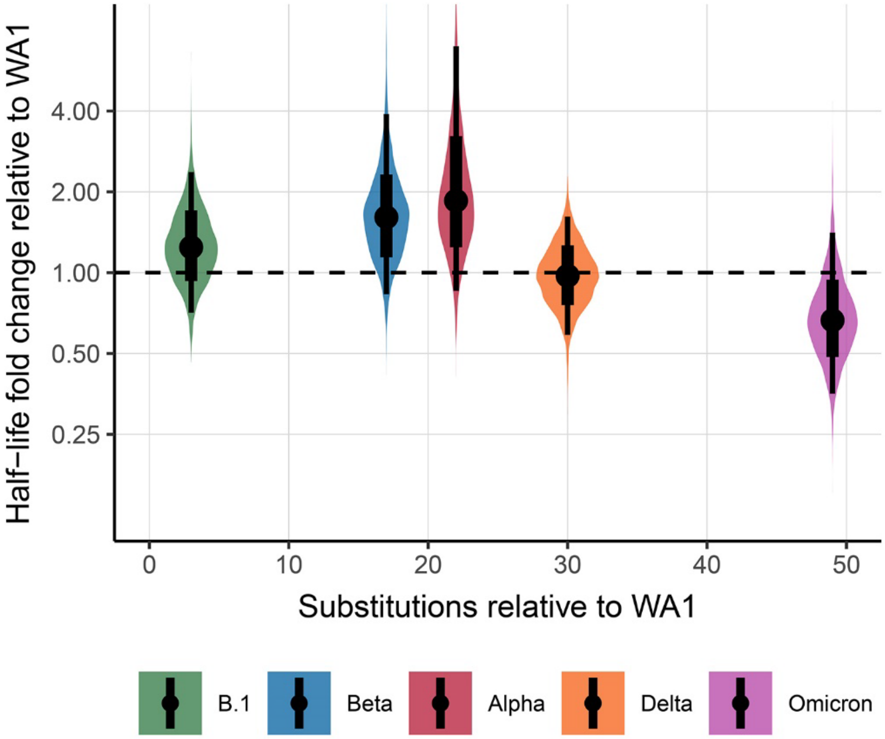

Appendix Figure 1. Variant half-lives as a function of amino acid divergence from the WA1 Lineage A variant. Violin plots show shape of the posterior distribution for the half-life, plotted on a logarithmic scale. Point shows the posterior median estimate, and black lines show a 68% (thick) and 95% (thin) credible interval.

A

Isolate	WHO Label	5	13	18	19	20	26	67	69	70	80	95	138	141	142	143
hu/USA/CA_CDC_5574/2020	-	L	S	L	T	T	P	A	H	V	D	T	D	L	G	V
hCoV-19/USA/MT-RML-7/2020	-	L	S	L	T	T	P	A	H	V	D	T	D	L	G	V
hCoV-19/England/204820464/2020	Alpha	L	S	L	T	T	P	A	DEL	DEL	D	T	D	L	G	V
hCoV-19/USA/MD-HP01542/2021	Beta	L	S	F	T	T	P	A	H	V	A	T	D	L	G	V
hCoV-19/USA/KY-CDC-2-4242084/20	Delta	L	S	L	R	T	P	A	H	V	D	T	D	L	G	V
hCoV-19/USA/WI-WSLH-221686/202	Omicron	L	S	L	T	T	P	V	DEL	DEL	D	I	D	L	D	DEL

Isolate	WHO Label	144	145	152	154	156	157	158	190	211	212	214	215	241	242	243	253	255	258
hu/USA/CA_CDC_5574/2020	-	Y	Y	W	E	E	F	R	R	N	L	R	D	L	L	A	D	S	W
hCoV-19/USA/MT-RML-7/2020	-	Y	Y	W	E	E	F	R	R	N	L	R	D	L	L	A	D	S	W
hCoV-19/England/204820464/2020	Alpha	DEL	Y	W	E	E	F	R	R	N	L	R	D	L	L	A	D	S	W
hCoV-19/USA/MD-HP01542/2021	Beta	Y	Y	W	E	E	F	R	R	N	L	R	G	DEL	DEL	DEL	D	S	W
hCoV-19/USA/KY-CDC-2-4242084/20	Delta	Y	Y	W	E	DEL	DEL	G	R	N	L	R	D	L	L	A	D	S	W
hCoV-19/USA/WI-WSLH-221686/202	Omicron	DEL	DEL	W	E	E	F	R	R	DEL	I	REPE	D	L	L	A	D	S	W

Isolate	WHO Label	339	346	371	373	375	417	440	446	452	477	478	484	493	496	498	501	505	547	570	614	655	677	679	681
hu/USA/CA_CDC_5574/2020	-	G	R	S	S	S	K	N	G	L	S	T	E	Q	G	Q	N	Y	T	A	D	H	Q	N	P
hCoV-19/USA/MT-RML-7/2020	-	G	R	S	S	S	K	N	G	L	S	T	E	Q	G	Q	N	Y	T	A	G	H	Q	N	P
hCoV-19/England/204820464/2020	Alpha	G	R	S	S	S	K	N	G	L	S	T	E	Q	G	Q	Y	Y	T	D	G	H	Q	N	H
hCoV-19/USA/MD-HP01542/2021	Beta	G	R	S	S	S	N	N	G	L	S	T	K	Q	G	Q	Y	Y	T	A	G	H	Q	N	P
hCoV-19/USA/KY-CDC-2-4242084/20	Delta	G	R	S	S	S	K	N	G	R	S	K	E	Q	G	Q	N	Y	T	A	G	H	Q	N	R
hCoV-19/USA/WI-WSLH-221686/202	Omicron	D	R	L	P	F	N	K	S	L	N	K	A	R	S	R	Y	H	K	A	G	Y	Q	K	H

S1

RBD

RBM

B

Isolate	WHO Label	701	716	764	796	856	859	888	950	954	957	969	981	982	1027	1071	1118	1176
hu/USA/CA_CDC_5574/2020	-	A	T	N	D	N	T	F	D	Q	Q	N	L	S	T	Q	D	V
hCoV-19/USA/MT-RML-7/2020	-	A	T	N	D	N	T	F	D	Q	Q	N	L	S	T	Q	D	V
hCoV-19/England/204820464/2020	Alpha	A	I	N	D	N	T	F	D	Q	Q	N	L	A	T	Q	H	V
hCoV-19/USA/MD-HP01542/2021	Beta	V	T	N	D	N	T	F	D	Q	Q	N	L	S	T	Q	D	V
hCoV-19/USA/KY-CDC-2-4242084/20	Delta	A	T	N	D	N	T	F	N	Q	Q	N	L	S	T	Q	D	V
hCoV-19/USA/WI-WSLH-221686/202	Omicron	A	T	K	Y	K	T	F	D	H	Q	K	F	S	T	Q	D	V

S2

Appendix Figure 2. Amino acid substitutions and deletions in the S1 and S2 regions of the spike protein for the Variants-of-Concern studied.

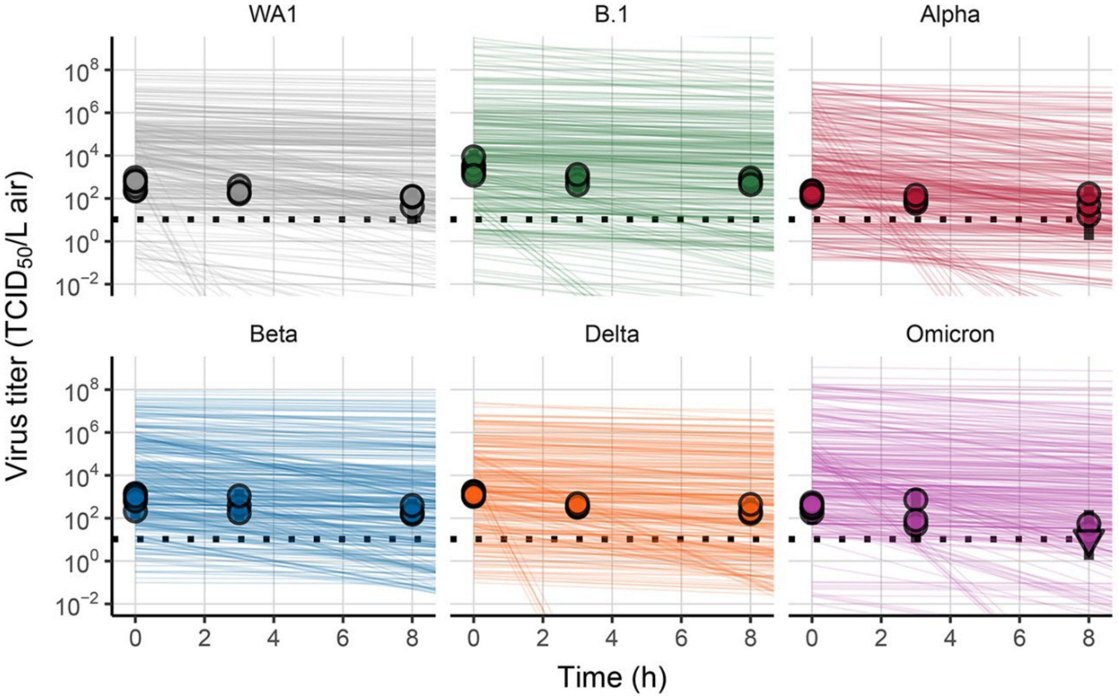

Appendix Figure 3. Prior predictive checks for aerosol experiments. Semi-transparent lines show random draws from the joint prior distribution for drum run intercepts ($t = 0$ values) and variant decay rates.We perform 50 random draws and then plot one line per draw for each drum run. This yields 300 plotted lines per variant. Points with black bars show individually-estimated titer values (point: posterior median titer estimate; bar: 95% credible interval). Points at 3 h and 8 h are shifted up or down by the physical / non-inactivation change in viral material estimated from qPCR data (see <u>Bayesian inference methods: qPCR adjustment for changes in sampled viral material</u>), to enable visual comparison with predicted decay (which reflects only inactivation effects). Samples with no positive titration wells are plotted as triangles at the approximate LOD (dotted horizontal line). Wide line coverage relative to data shows that model considered many possible decay kinetics as a priori plausible.

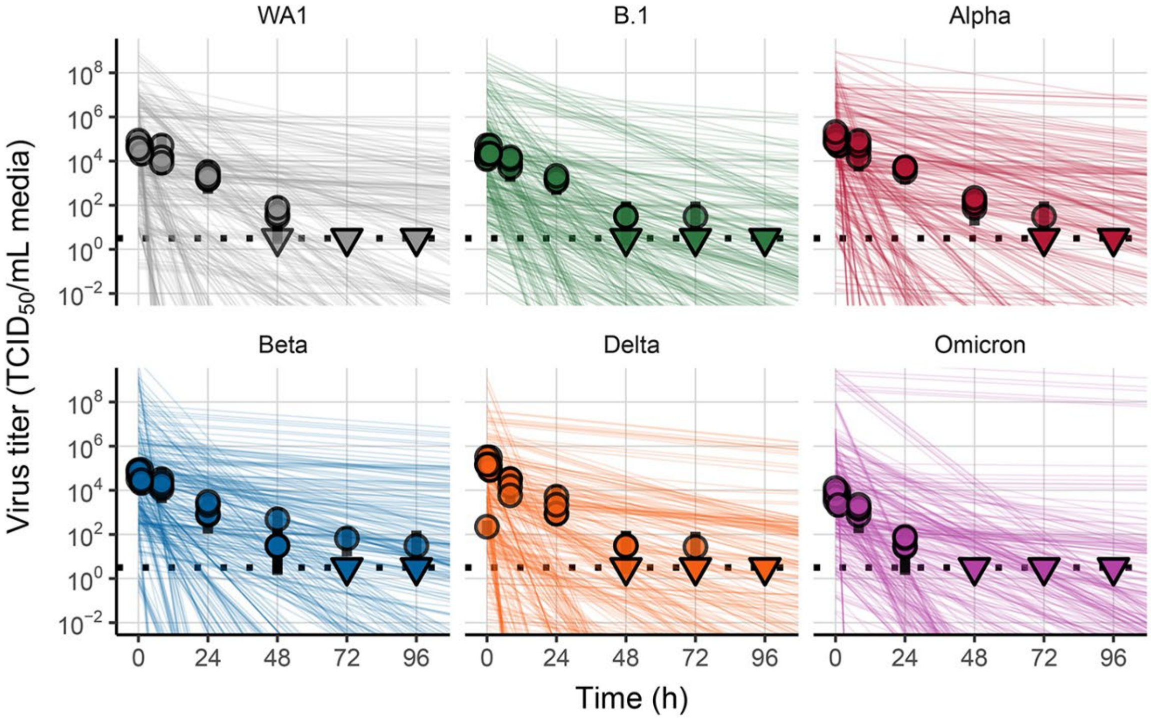

Appendix Figure 4. Prior predictive checks for surface experiments. Semi-transparent lines show random draws from the joint prior distribution for titer intercepts ($t = 0$ values) and variant decay rates. We perform 50 random draws and then plot 6 random initial titers per draw for each variant. This yields 300 plotted lines per variant. We choose a new group of 6 random initial titers for each new draw-variant pair. Points with black bars show individually estimated titer values (point: posterior median titer estimate; bar: 95% credible interval). Samples with no positive titration wells are plotted as triangles at the approximate LOD (dotted horizontal line). Wide line coverage relative to data shows that model considered many possible decay kinetics as a priori plausible.

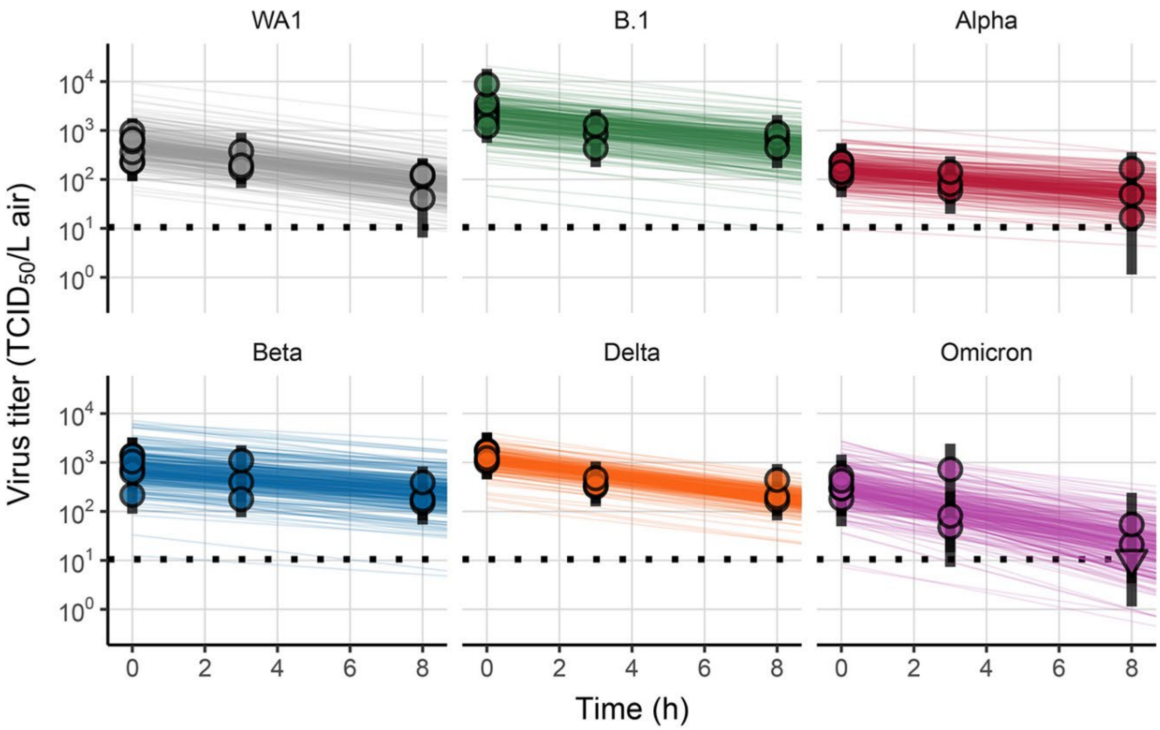

Appendix Figure 5. Posterior predictive checks for aerosol experiments. Semi-transparent lines show random draws from the joint posterior predictive distribution for drum run intercepts ($t = 0$ values) and variant decay rates.We perform 50 random draws and then plot one line per draw for each drum run. This yields 300 plotted lines per variant. Points with black bars show individually-estimated titer values (point: posterior median titer estimate; bar: 95% credible interval). Points at 3 h and 8 h are shifted up or down by the physical / non-inactivation change in viral material estimated from qPCR data (see <u>Bayesian inference methods: qPCR adjustment for changes in sampled viral material</u>), to enable visual comparison with predicted decay (which reflects only inactivation effects). Samples with no positive titration wells are plotted as triangles at the approximate LOD (dotted horizontal line). Tight fit of lines to data suggests that hierarchical model of intercepts and estimated exponential decay kinetics for infectious virus describe the data well.

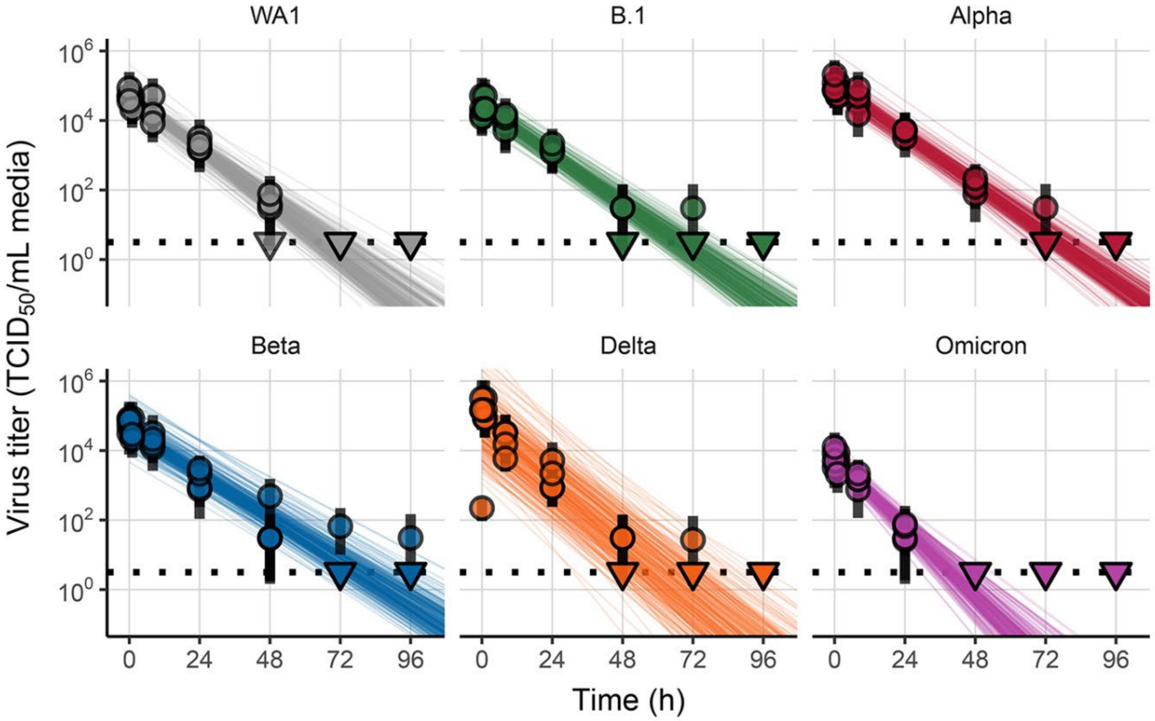

Appendix Figure 6. Posterior predictive checks for surface experiments. Semi-transparent lines show random draws from the joint posterior predictive distribution for titer intercepts ($t = 0$ values) and variant decay rates. We perform 50 random draws and then plot 6 random initial titers per draw for each variant. This yields 300 plotted lines per variant. We choose a new group of 6 random initial titers for each new draw-variant pair. Points with black bars show individually-estimated titer values (point: posterior median titer estimate; bar: 95% credible interval). Samples with no positive titration wells are plotted as triangles at the approximate LOD (dotted horizontal line). Tight fit of lines to data suggests that hierarchical model of intercepts and estimated exponential decay kinetics for infectious virus describe the data well.

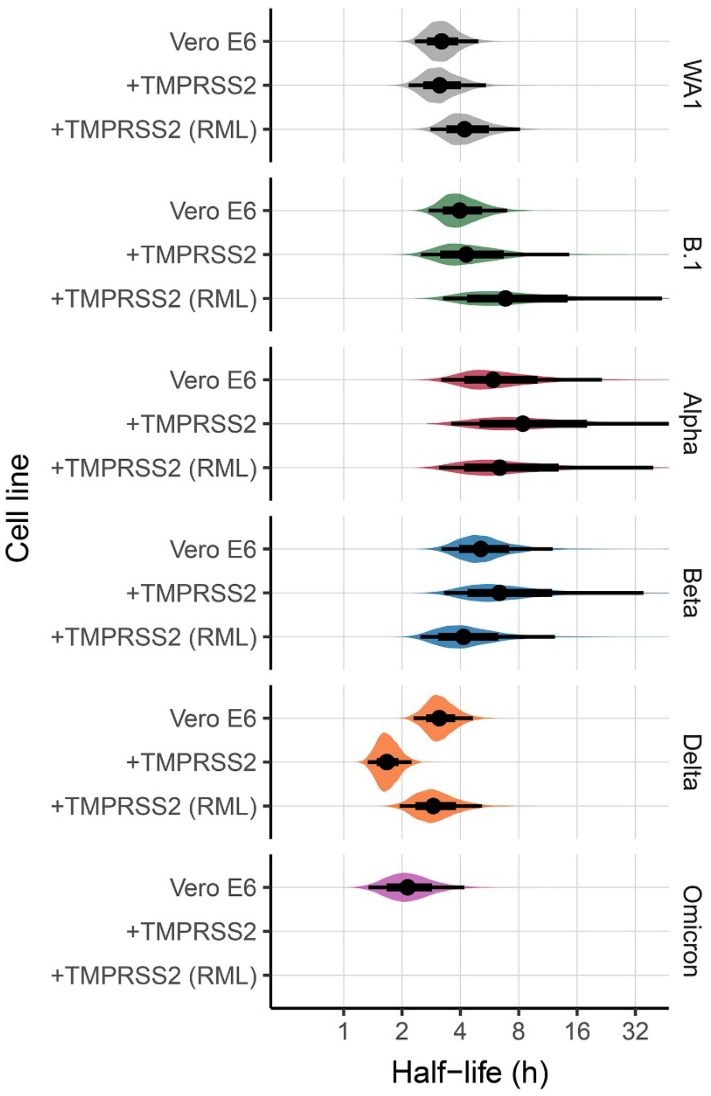

Appendix Figure 7. Estimated half-lives of infectious virus in aerosols by variant and cell line. “Vero E6” denotes standard Vero E6 cell titration as plotted in Main Text; “+TMPRSS2” denotes Vero E6-TMPRSS2-T2A-ACE2 cells; “+TMPRSS2 (RML)” denotes Vero-TMPRSSII-RML cells. Half-lives plotted on a logarithmic scale. Violin plots show approximate shape of the posterior distribution. Point shows the posterior median estimate and lines show a 68% (thick) and 95% (thin) credible interval. Pattern across variants is consistent regardless of cell line. Delta shows somewhat shorter half-life on one line of TMPRSS2 expressing cells.

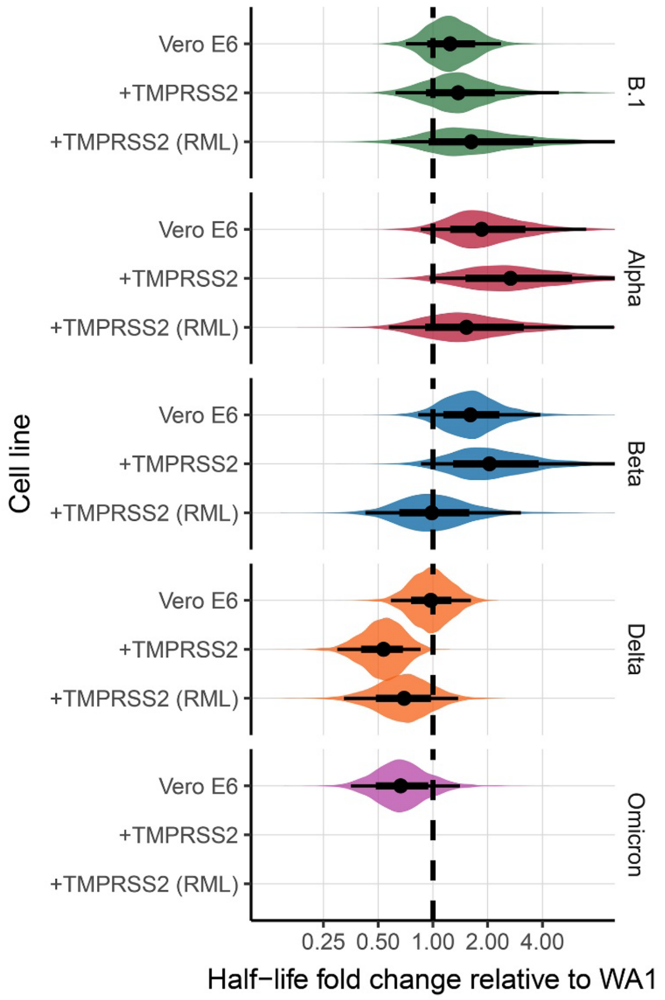

Appendix Figure 8. Estimated fold-change in infectious virus half-life in aerosols relative to WA1 by variant and cell line. "Vero E6" denotes standard Vero E6 cell titration as plotted in Main Text; "+TMPRSS2" denotes Vero E6-TMPRSS2-T2A-ACE2 cells; "+TMPRSS2 (RML) denotes Vero-TMPRSSII-RML cells. Fold-changes plotted on a logarithmic scale centered on 1 (no change), which is indicated by a dashed line. Violin plots show approximate shape of the posterior distribution. Point shows the posterior median estimate and lines show a 68% (thick) and 95% (thin) credible interval. Pattern across variants is consistent regardless of cell line. Delta shows a greater reduction in half-life relative to WA1 on one line of TMPRSS2-expressing cells, corresponding to its substantially lower measured half-life on that cell line, see Appendix Figure 7.

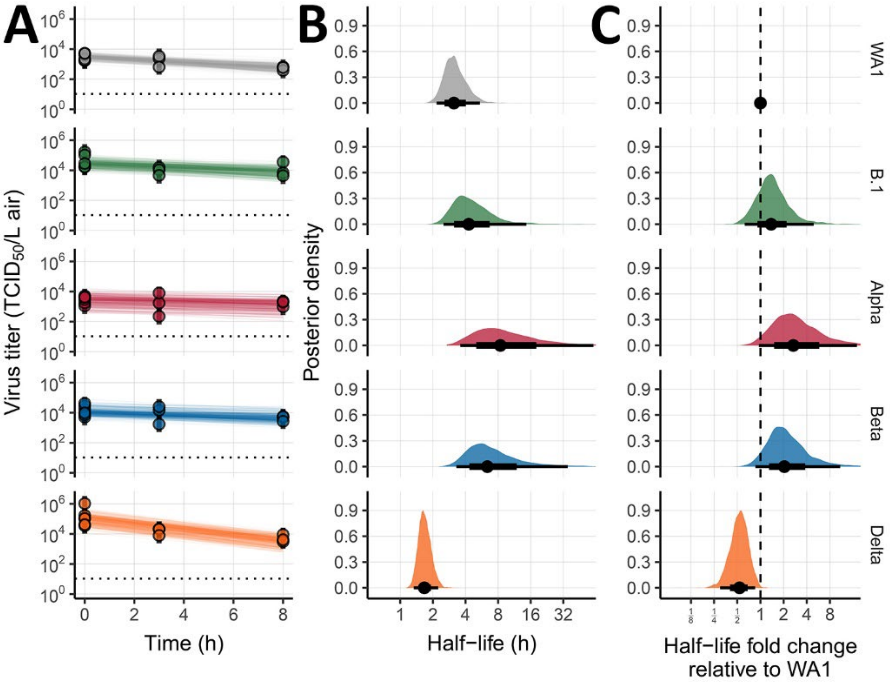

Appendix Figure 9. Version of Main Text Figure 1 with titration on modified Vero cells expressing TMPRSS2 (Vero E6-TMPRSS2-T2A-ACE2). **A**: Regression lines representing predicted exponential decay of $\log_{10}$ virus titer over time compared to measured (directly-inferred) virus titers. Points with black bars show individually-estimated titer values (point: posterior median titer estimate; bar: 95% credible interval). Points at 3 h and 8 h are shifted up or down by the physical / non-inactivation change in viral material estimated from qPCR data (see Bayesian inference methods: qPCR adjustment for changes in sampled viral material), to enable visual comparison with predicted decay (which reflects only inactivation effects). Semi-transparent lines show random draws from the joint posterior distribution of the exponential decay rate and the drum run intercept (virus titer at $t = 0$); this visualizes the range of plausible decay patterns for each experimental condition. We perform 50 random draws and then plot one line per draw for each drum run. This yields 300 plotted lines per variant. **B**: Inferred virus half-lives by variant, plotted on a logarithmic scale. Density plots show the shape of the posterior distribution. Dots show the posterior median half-life estimate and black lines show a 68% (thick) and 95% (thin) credible interval. **C**: Inferred ratio of variant virus half-lives to that of WA1 (fold-change), plotted on a logarithmic scale and centered on 1 (no change, dashed line). Dot shows the posterior median estimate and black lines show a 68% (thick) and 95% (thin) credible interval.

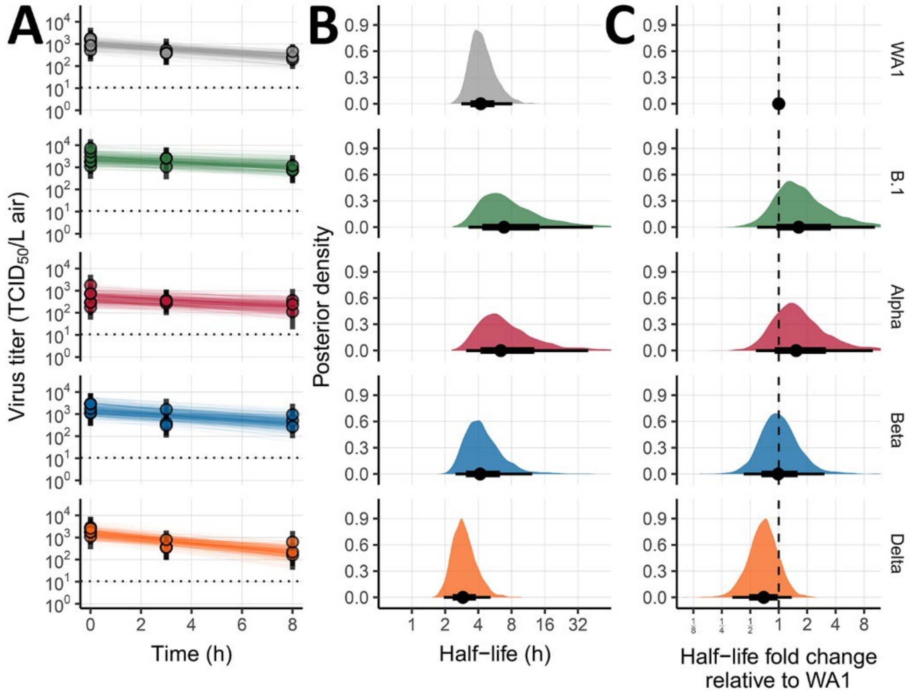

Appendix Figure 10. Version of Main Text Figure 1 with titration on modified Vero cells expressing TMPRSS2 (Vero-TMPRSSII-RML). **A**: Regression lines representing predicted exponential decay of $\log_{10}$ virus titer over time compared to measured (directly-inferred) virus titers. Points with black bars show individually-estimated titer values (point: posterior median titer estimate; bar: 95% credible interval). Points at 3 h and 8 h are shifted up or down by the physical / non-inactivation change in viral material estimated from qPCR data (see Bayesian inference methods: qPCR adjustment for changes in sampled viral material), to enable visual comparison with predicted decay (which reflects only inactivation effects). Semi-transparent lines show random draws from the joint posterior distribution of the exponential decay rate and the drum run intercept (virus titer at $t = 0$); this visualizes the range of plausible decay patterns for each experimental condition. We perform 50 random draws and then plot one line per draw for each drum run. This yields 300 plotted lines per variant. **B**: Inferred virus half-lives by variant, plotted on a logarithmic scale. Density plots show the shape of the posterior distribution. Dots show the posterior median half-life estimate and black lines show a 68% (thick) and 95% (thin) credible interval. **C**: Inferred ratio of variant virus half-lives to that of WA1 (fold-change), plotted on a logarithmic scale and centered on 1 (no change, dashed line). Dot shows the posterior median estimate and black lines show a 68% (thick) and 95% (thin) credible interval.

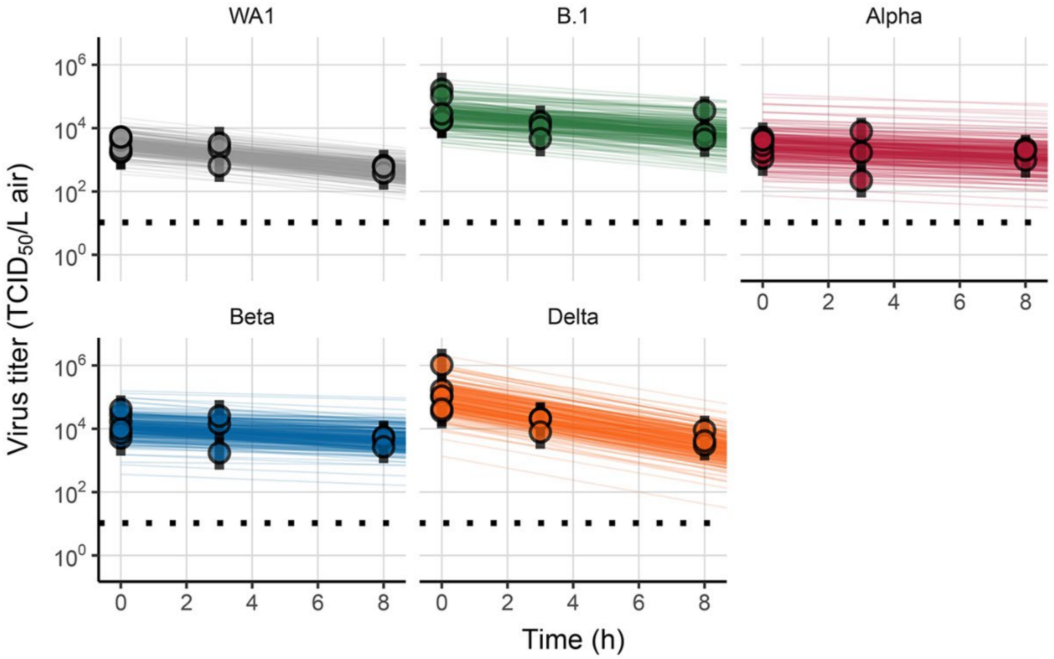

Appendix Figure 11. Posterior predictive checks for aerosol experiments with titration on modified Vero cells expressing TMPRSS2 (Vero E6-TMPRSS2-T2A-ACE2). Semi-transparent lines show random draws from the joint posterior predictive distribution for drum run intercepts ($t = 0$ values) and variant decay rates.We perform 50 random draws and then plot one line per draw for each drum run. This yields 300 plotted lines per variant. Points with black bars show individually-estimated titer values (point: posterior median titer estimate; bar: 95% credible interval). Points at 3 h and 8 h are shifted up or down by the physical / non-inactivation change in viral material estimated from qPCR data (see Bayesian inference methods: qPCR adjustment for changes in sampled viral material), to enable visual comparison with predicted decay (which reflects only inactivation effects). Samples with no positive titration wells are plotted as triangles at the approximate LOD (dotted horizontal line). Tight fit of lines to data suggests that hierarchical model of intercepts and estimated exponential decay kinetics for infectious virus describe the data well.

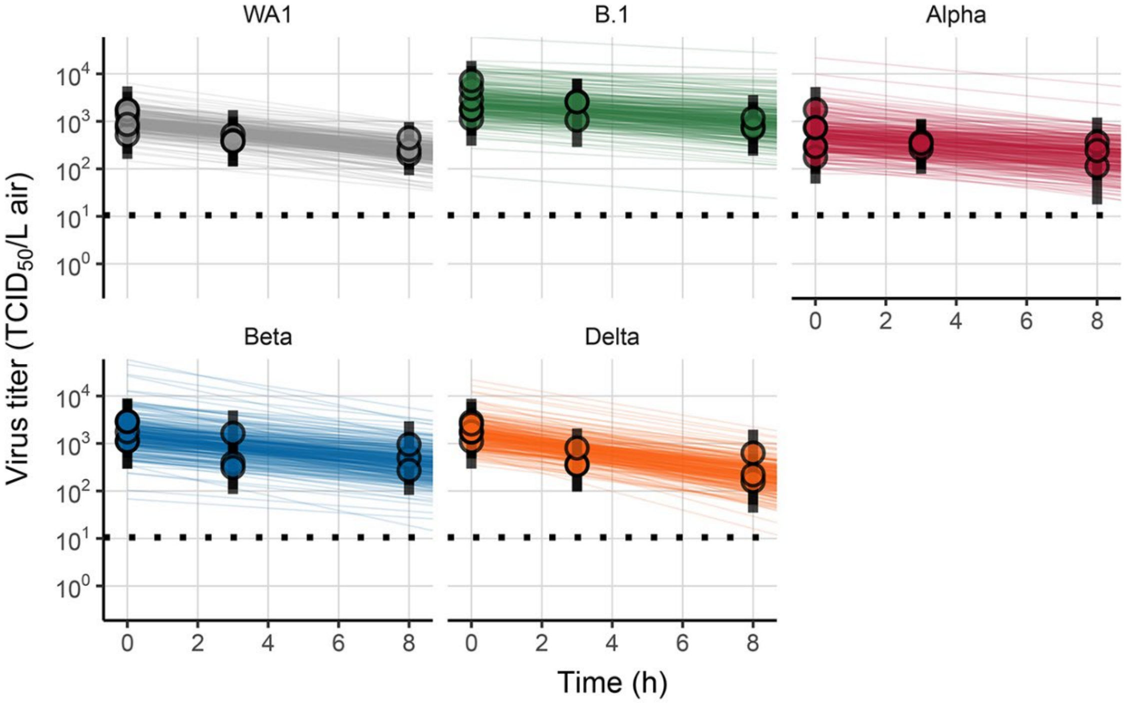

Appendix Figure 12. Posterior predictive checks for aerosol experiments with titration on modified Vero cells expressing TMPRSS2 (Vero-TMPRSSII-RML. Semi-transparent lines show random draws from the joint posterior predictive distribution for drum run intercepts ($t = 0$ values) and variant decay rates.We perform 50 random draws and then plot one line per draw for each drum run. This yields 300 plotted lines per variant. Points with black bars show individually-estimated titer values (point: posterior median titer estimate; bar: 95% credible interval). Points at 3 h and 8 h are shifted up or down by the physical / non-inactivation change in viral material estimated from qPCR data (see Bayesian inference methods: qPCR adjustment for changes in sampled viral material), to enable visual comparison with predicted decay (which reflects only inactivation effects). Samples with no positive titration wells are plotted as triangles at the approximate LOD (dotted horizontal line). Tight fit of lines to data suggests that hierarchical model of intercepts and estimated exponential decay kinetics for infectious virus describe the data well.

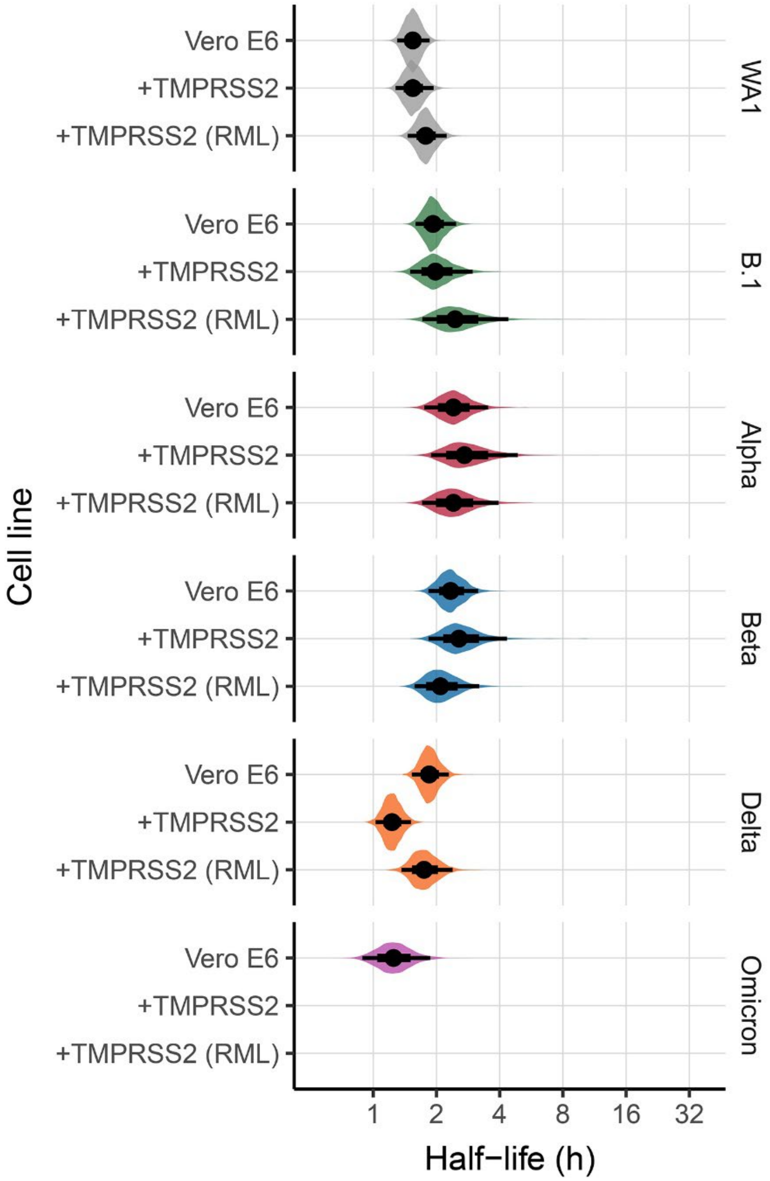

Appendix Figure 13. Estimated half-lives of infectious virus in aerosols by variant and cell line, no qPCR adjustment performed. Half-lives plotted on a logarithmic scale. Violin plots show approximate shape of the posterior distribution. Point shows the posterior median estimate and lines show a 68% (thick) and 95% (thin) credible interval.

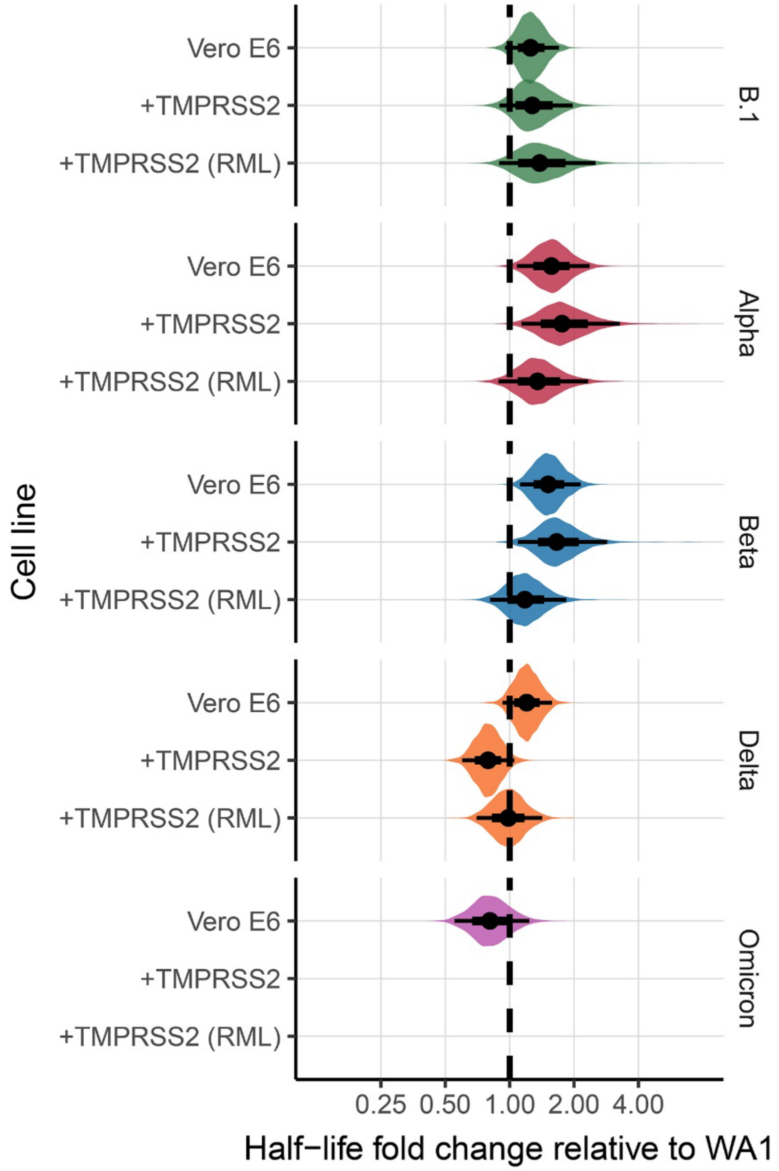

Appendix Figure 14. Estimated fold-change in infectious virus half-life in aerosols relative to WA1 by variant and cell line, no qPCR adjustment performed. Fold-changes plotted on a logarithmic scale centered on 1 (no change), which is indicated by a dashed line. Violin plots show approximate shape of the posterior distribution. Point shows the posterior median estimate and lines show a 68% (thick) and 95% (thin) credible interval.

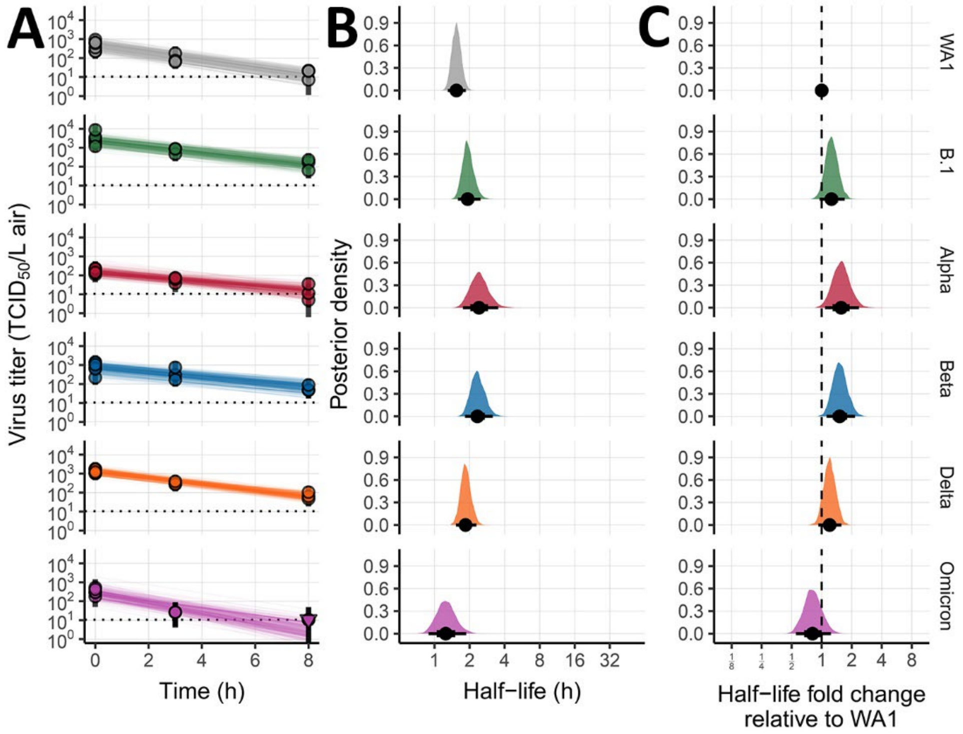

Appendix Figure 15. Version of Main Text Figure 1 with titration on standard Vero E6 cells, no qPCR adjustment performed. **A**: Regression lines representing predicted exponential decay of $\log_{10}$ virus titer over time compared to measured (directly-inferred) virus titers. Points with black bars show individually-estimated titer values (point: posterior median titer estimate; bar: 95% credible interval). Samples with no positive titration wells are plotted as triangles at the approximate LOD (dotted horizontal line). Semi-transparent lines show random draws from the joint posterior distribution of the exponential decay rate and the drum run intercept (virus titer at $t = 0$); this visualizes the range of plausible decay patterns for each experimental condition. We perform 50 random draws and then plot one line per draw for each drum run. This yields 300 plotted lines per variant. **B**: Inferred virus half-lives by variant, plotted on a logarithmic scale. Density plots show the shape of the posterior distribution. Dots show the posterior median half-life estimate and black lines show a 68% (thick) and 95% (thin) credible interval. **C**: Inferred ratio of variant virus half-lives to that of WA1 (fold-change), plotted on a logarithmic scale and centered on 1 (no change, dashed line). Dot shows the posterior median estimate and black lines show a 68% (thick) and 95% (thin) credible interval.

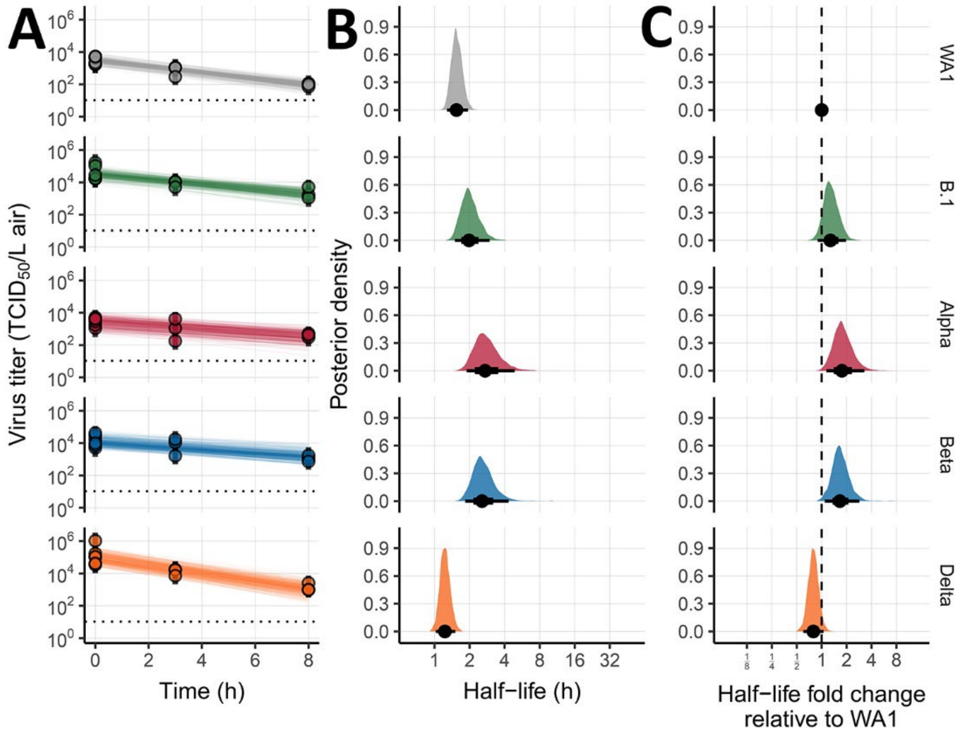

Appendix Figure 16. Version of Main Text Figure 1 with titration on modified Vero cells expressing TMPRSS2 (Vero E6-TMPRSS2-T2A-ACE2), no qPCR adjustment performed. **A**: Regression lines representing predicted exponential decay of $\log_{10}$ virus titer over time compared to measured (directly-inferred) virus titers. Points with black bars show individually-estimated titer values (point: posterior median titer estimate; bar: 95% credible interval). Dashed horizontal line shows approximate LOD for individual titers. Semi-transparent lines show random draws from the joint posterior distribution of the exponential decay rate and the drum run intercept (virus titer at $t = 0$); this visualizes the range of plausible decay patterns for each experimental condition. We perform 50 random draws and then plot one line per draw for each drum run. This yields 300 plotted lines per variant. **B**: Inferred virus half-lives by variant, plotted on a logarithmic scale. Density plots show the shape of the posterior distribution. Dots show the posterior median half-life estimate and black lines show a 68% (thick) and 95% (thin) credible interval. **C**: Inferred ratio of variant virus half-lives to that of WA1 (fold-change), plotted on a logarithmic scale and centered on 1 (no change, dashed line). Dot shows the posterior median estimate and black lines show a 68% (thick) and 95% (thin) credible interval.

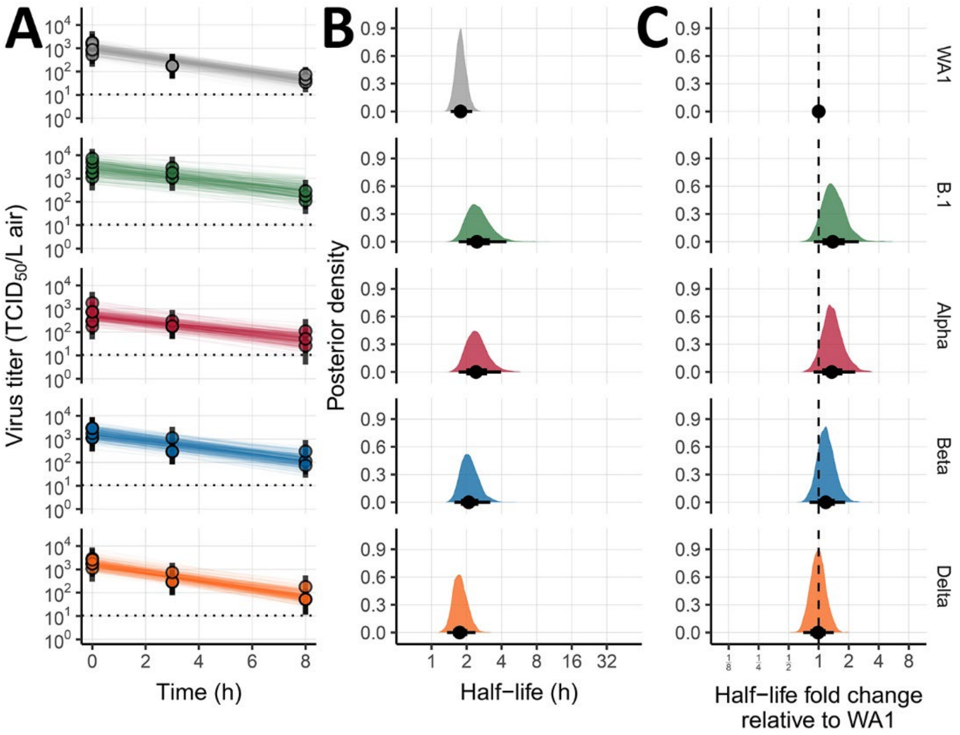

Appendix Figure 17. Version of Main Text Figure 1 with titration on modified Vero cells expressing TMPRSS2 (Vero-TMPRSSII-RML), no qPCR adjustment performed. **A**: Regression lines representing predicted exponential decay of $\log_{10}$ virus titer over time compared to measured (directly inferred) virus titers. Points with black bars show individually estimated titer values (point: posterior median titer estimate; bar: 95% credible interval). Dashed horizontal line shows approximate LOD for individual titers. Semi-transparent lines show random draws from the joint posterior distribution of the exponential decay rate and the drum run intercept (virus titer at $t = 0$); this visualizes the range of plausible decay patterns for each experimental condition. We perform 50 random draws and then plot one line per draw for each drum run. This yields 300 plotted lines per variant. **B**: Inferred virus half-lives by variant, plotted on a logarithmic scale. Density plots show the shape of the posterior distribution. Dots show the posterior median half-life estimate and black lines show a 68% (thick) and 95% (thin) credible interval. **C**: Inferred ratio of variant virus half-lives to that of WA1 (fold-change), plotted on a logarithmic scale and centered on 1 (no change, dashed line). Dot shows the posterior median estimate and black lines show a 68% (thick) and 95% (thin) credible interval.
